# Optimizing Attenuation Correction in ^68^Ga-PSMA PET Imaging Using Deep Learning and Artifact-Free Dataset Refinement

**DOI:** 10.3390/diagnostics15111400

**Published:** 2025-05-31

**Authors:** Masoumeh Dorri Giv, Guluzar Ozbolat, Hossein Arabi, Somayeh Malmir, Shahrokh Naseri, Vahid Roshan Ravan, Hossein Akbari-Lalimi, Raheleh Tabari Juybari, Ghasem Ali Divband, Nasrin Raeisi, Vahid Reza Dabbagh Kakhki, Emran Askari, Sara Harsini

**Affiliations:** 1 Nuclear Medicine Research Center, Department of Nuclear Medicine, Ghaem Hospital, Mashhad University of Medical Science, Mashhad 6541747187, Iran; dorrigm981@mums.ac.ir (M.D.G.); vdrn42@gmail.com (V.R.R.); reisien4001@mums.ac.ir (N.R.); emran.a69@gmail.com (E.A.); 2 Faculty of Health Science, Sinop University, Sinop 57000, Turkey; guluzarbolat@gmail.com; 3 Division of Nuclear Medicine and Molecular Imaging, Department of Medical Imaging, Geneva University Hospital, CH-1211 Geneva, Switzerland; hossein.arabi@unige.ch; 4 Department of Physics, Payame Noor University, Tehran 193954697, Iran; 5 Department of Medical Physics, Faculty of Medicine, Mashhad University of Medical Science, Mashhad 9177948564, Iran; naserish@mums.ac.ir; 6 Department of Medical Physics and Radiology, School of Allied Medical Sciences, Gonabad University of Medical Sciences, Gonabad 8317785741, Iran; h_akbari_l@yahoo.com; 7 Department of Radiology Technology, Behbahan Faculty of Medical Science, Behbahan 6361796819, Iran; r.tabari@gmail.com; 8 Department of Nuclear Medicine, Jam Hospital, Tehran 1588657915, Iran; divband_ali@yahoo.com; 9 Department of Molecular Imaging and Therapy, BC Cancer Research Institute, Vancouver, BC V5Z 1L3, Canada

**Keywords:** attenuation correction, positron emission tomography computed tomography, deep learning, image artifacts, neural networks

## Abstract

**Background/Objectives:** Attenuation correction (AC) is essential for achieving quantitatively accurate PET imaging. In ^68^Ga-PSMA PET, however, artifacts such as respiratory motion, halo effects, and truncation errors in CT-based AC (CT-AC) images compromise image quality and impair model training for deep learning-based AC. This study proposes a novel artifact-refinement framework that filters out corrupted PET-CT images to create a clean dataset for training an image-domain AC model, eliminating the need for anatomical reference scans. **Methods:** A residual neural network (ResNet) was trained using paired PET non-AC and PET CT-AC images from a dataset of 828 whole-body ^68^Ga-PSMA PET-CT scans. An initial model was trained using all data and employed to identify artifact-affected samples via voxel-level error metrics. These outliers were excluded, and the refined dataset was used to retrain the model with an L2 loss function. Performance was evaluated using metrics including mean error (ME), mean absolute error (MAE), relative error (RE%), RMSE, and SSIM on both internal and external test datasets. **Results:** The model trained with the artifact-free dataset demonstrated significantly improved performance: ME = −0.009 ± 0.43 SUV, MAE = 0.09 ± 0.41 SUV, and SSIM = 0.96 ± 0.03. Compared to the model trained on unfiltered data, the purified data model showed enhanced quantitative accuracy and robustness in external validation. **Conclusions:** The proposed data purification framework significantly enhances the performance of deep learning-based AC for ^68^Ga-PSMA PET by mitigating artifact-induced errors. This approach facilitates reliable PET imaging in the absence of anatomical references, advancing clinical applicability and image fidelity.

## 1. Introduction

Nuclear medicine is a specialized field of medical imaging that utilizes radioactive substances for the diagnosis and treatment of disease. Among its core imaging modalities are single-photon emission computed tomography (SPECT) and positron emission tomography (PET), which primarily focus on assessing physiological function rather than anatomical structures. For this reason, these techniques are often referred to as physiological imaging modalities. When combined with anatomical imaging techniques such as computed tomography (CT) and magnetic resonance imaging (MRI), nuclear medicine provides comprehensive maps of both normal and pathological tissue distribution, enabling accurate diagnosis, staging, and monitoring of disease [1,2].

Currently, approximately one-third of medical imaging and therapeutic procedures in modern healthcare involve the use of ionizing radiation or radioactive tracers. Given the widespread clinical use of hybrid anatomical/functional imaging, there is a growing demand for improvements in image quality, reduction in scan time and radiation exposure, enhancement of diagnostic algorithms, and development of robust, quantitative imaging methods. These improvements are essential for more accurate therapy guidance, reduced patient burden, lower operational costs, and better clinical outcomes. Addressing these challenges requires innovative technologies and methodologies to advance nuclear medicine and, more broadly, support public health efforts [3]. While general PET imaging faces challenges such as motion artifacts and truncation errors, the use of ^68^Ga-labeled Prostate-Specific Membrane Antigen (PSMA) tracers for prostate cancer imaging introduces additional complexities. Specifically, ^68^Ga-PSMA PET imaging is prone to halo artifacts around high-uptake regions like the kidneys and bladder, which can mask adjacent lesions, and respiratory motion artifacts that may misalign PET and CT images, particularly at the thoracoabdominal interface. Furthermore, truncation errors in patients with larger body sizes and metallic artifacts from implants can further degrade image quality. These issues underscore the need for advanced attenuation correction techniques tailored to the unique characteristics of ^68^Ga-PSMA PET imaging, ensuring that this modality can fulfill its promise of improved diagnostic accuracy for prostate cancer [4,5].

In parallel with the clinical adoption of PSMA-targeted imaging, several technological advances have contributed to improved PET/CT image quality. Time-of-Flight (TOF) techniques enhance lesion localization by using timing information from photon pairs, while Point Spread Function (PSF) modeling refines spatial resolution. Resolution modeling is of paramount importance in ^68^Ga-PSMA PET imaging due to the higher positron range of Ga-68 compared to ^18^F-FDG PET imaging. Additionally, modern iterative CT reconstruction algorithms, including model-based and deep learning-assisted approaches, have been employed to reduce noise, minimize artifacts, and lower radiation dose. While these methods improve image fidelity, they do not fully eliminate motion-related and scatter-induced distortions, especially in high-contrast studies such as ^68^Ga-PSMA PET. These limitations emphasize the need for complementary solutions like deep learning-based attenuation correction.

In ^68^Ga-PSMA PET/MRI imaging, the uptake of PSMA in abdominal fat and soft tissue is minimal, while most of the tracer is excreted through the urinary system and accumulates in the kidneys and bladder. This results in a stark contrast in activity concentration between these organs and the surrounding tissues. The resulting high organ-to-background ratio (OBR) often produces photopenic artifacts—commonly referred to as halo artifacts—particularly around the kidneys and bladder, more noticeably in PET/MRI than in PET/CT.

These halo artifacts pose significant challenges to the clinical application of ^68^Ga-PSMA PET/MRI in prostate cancer detection and staging. Lesions located near the bladder, including primary tumors and local recurrences, may be concealed or exhibit altered tracer uptake due to the artifact, thus compromising diagnostic accuracy. Similarly, metastases in the retroperitoneal region can be masked, impairing tumor detection and staging performance. Studies suggest that these artifacts are primarily attributable to inadequate scatter correction algorithms [6,7].

Quantitative and semi-quantitative measures from PET, such as the standardized uptake value (SUV), are vital for evaluating disease and treatment response. However, their accuracy is heavily influenced by physical image-degrading effects—especially photon attenuation and Compton scatter. The latter, resulting from photon interactions with dense materials (e.g., patient tissues, scanner bed), contributes significantly to signal distortion—up to 30–35% in brain PET and 50–60% in whole-body PET imaging [8,9]. Although photons scattered outside the energy window are excluded during scatter correction, they can still impact attenuation correction and compromise image fidelity [9].

Attenuation correction (AC) and scatter correction (SC) are indispensable for accurate PET quantification. Conventional AC methods employ CT-derived attenuation maps, which convert Hounsfield units into linear attenuation coefficients at 511 keV [10]. Despite their widespread use, these methods are limited by issues such as additional radiation dose, misalignment errors, and incompatibility with MRI data, which lack direct electron density information [11]. Misassigned lines of response (LORs) due to scatter-induced path deviations further necessitate improved SC algorithms [9,11].

Initial efforts to perform AC and SC directly in the image domain focused on brain PET, benefiting from the structural stability and uniformity across patients [12,13]. Recently, there has been a push toward adapting these methods for whole-body PET imaging, showing promising accuracy even with small datasets [14]. However, broader application remains challenging due to anatomical diversity, variable posture, and respiratory motion—all of which introduce variability and potential outliers in the data. Addressing these factors is crucial for achieving robust AC across whole-body scans.

In this context, deep learning has emerged as a powerful solution, offering transformative advances in medical imaging. Applications include image denoising, resolution enhancement, and cross-modality transformations [15,16]. Deep learning models have also shown promise in performing AC directly in the image domain [17,18], bypassing the need for anatomical reference images [3,19]. However, the presence of artifacts—particularly in PET/CT attenuation-corrected images—can impair model performance. Artifacts such as respiratory motion, truncation, and halo effects can distort tracer distribution and introduce quantification bias [9,10].

This study presents a novel artifact-refinement framework to address these issues. By automatically identifying and excluding corrupted PET-CT-AC images from the training dataset, we aim to develop a robust deep learning-based AC model trained on clean, artifact-free data. The model performs attenuation correction directly in the image domain and is specifically designed for whole-body ^68^Ga-PSMA PET imaging. By systematically managing outliers and accounting for respiratory motion, our approach enhances AC accuracy and improves generalizability.

Through rigorous error analysis and state-of-the-art deep learning techniques, this study contributes a practical and effective solution to one of the key limitations in quantitative PET imaging—namely, the presence of artifacts in training data. Our findings support the broader implementation of artifact-free, AI-based attenuation correction as a step toward more accurate, reliable, and clinically applicable PET imaging.

## 2. Materials and Methods

### 2.1. Dataset

This retrospective study utilized whole-body PET/CT imaging data collected from patients referred to Razavi Hospital (Mashhad, Iran) for prostate cancer evaluation between 2018 and 2022. The study was conducted in accordance with institutional ethical standards and received approval from the Institutional Review Board of Razavi Hospital. Informed consent was obtained from all participants prior to their inclusion.

A total of 828 whole-body ^68^Ga-PSMA PET/CT scans were included in the dataset. Among these, 784 scans were used for model training and 50 scans were reserved for testing purposes. The dataset comprised both artifact-free and artifact-affected images, including common issues such as halo artifacts, respiratory motion artifacts, and truncation artifacts, which are typically encountered in clinical PET/CT imaging.

All patients received an intravenous injection of 185 ± 21 MBq of ^68^Ga-PSMA radiotracer, and imaging was initiated 60 min post-injection. Low-dose CT scans were performed for attenuation correction using standardized acquisition protocols to ensure reproducibility and consistency across the dataset.

### 2.2. Data Acquisition

All PET/CT imaging procedures were conducted using a Biograph 6 TruePoint PET/CT scanner (Siemens Medical Solutions, six-slice). Prior to scanning, each patient’s medical history—including previous treatments such as prostatectomy or external beam radiation therapy—was reviewed to ensure that the imaging protocol was appropriately individualized.

Patients received an intravenous injection of ^68^Ga-PSMA at a dose of 2 MBq/kg. Imaging commenced 60 min post-injection and extended from the head to the mid-thigh to adequately capture anatomical regions relevant to prostate cancer diagnosis and staging. Before the PET acquisition, low-dose CT scans were obtained for attenuation correction using a six-slice helical CT scanner (Siemens Medical Solutions USA, Inc., Malvern, PA, USA). The CT acquisition parameters included a tube voltage of 110 kVp, a tube current of 52 mAs, and a slice thickness of 4 mm.

PET scans were acquired using six to eight bed positions, with an acquisition time of 3 min per bed position. Both attenuation and scatter-corrected PET images (PET-CTAC) and non-attenuation-corrected PET images (PET-non-AC) were reconstructed using the ordered-subsets expectation maximization (OSEM) algorithm. The reconstruction parameters consisted of two iterations, eight subsets, and the application of a 5 mm post-reconstruction Gaussian filter. The final image matrix size was 168 × 168, with a voxel size of 4.07 × 4.07 × 3 mm^3^.

This standardized acquisition and reconstruction protocol ensured high-quality, consistent imaging across all cases, providing a reliable foundation for model training and analysis.

### 2.3. Model Architecture

In this study, we adopted a deep learning-based attenuation correction (AC) approach utilizing a Residual Neural Network (ResNet). The primary objective was to develop an image-domain AC model for ^68^Ga-PSMA PET imaging, with improved robustness by training exclusively on artifact-free data.

**Figure 1:**
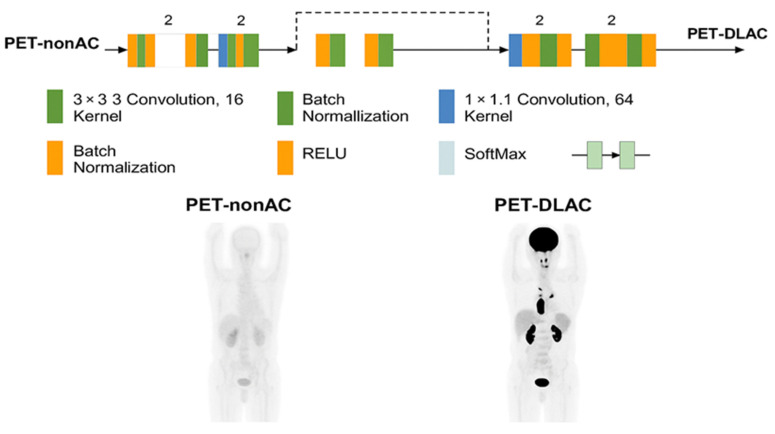
Architecture of the ResNet-based network used in this study, implemented via the NiftyNet platform. The model includes sequential 3D convolutional layers, batch normalization, ReLU activations, and residual blocks. Skip connections and SoftMax output layers further support optimization and classification robustness.

A 3D ResNet implemented in the NiftyNet platform, was employed specifically for image-domain attenuation correction in ^68^Ga-PSMA PET imaging. The architecture comprises 20 convolutional layers organized into residual blocks to facilitate robust feature learning and mitigate the vanishing gradient problem. The initial seven layers utilize 3 × 3 × 3 convolutional kernels to extract low-level features, such as edges and textures, from the input PET-non-AC images. The subsequent 13 layers employ dilated convolutions with dilation rates of 2 and 4 to capture mid- and high-level contextual information across a wider receptive field, which is critical for handling the anatomical variability and artifacts prevalent in whole-body PET imaging. Residual connections are integrated every two layers to enhance gradient flow and improve convergence, while skip connections further support optimization by preserving spatial information. Batch normalization and ReLU activation layers are interleaved throughout the network to stabilize training and introduce non-linearity. The model preserves the input resolution (168 × 168 × 3 mm^3^ voxel size) throughout, enabling voxel-wise predictions for direct mapping from PET-non-AC to PET-CT-AC images. A SoftMax output layer is used to ensure robust classification and intensity prediction, as illustrated in Figure 1 of the manuscript.

Model training was conducted in two stages to ensure robustness and mitigate the impact of artifacts in the dataset. In the first stage, the model was trained on the complete dataset of 828 whole-body ^68^Ga-PSMA PET/CT scans, using PET-non-AC images as input and PET-CT-AC images as ground truth. An L1 loss function was employed to reduce sensitivity to outliers caused by artifacts such as halo effects, respiratory motion, and truncation errors. Following this initial training, the model performed self-inference on the entire dataset, and voxel-wise error metrics (absolute relative error, mean absolute error, and root mean square error) were computed to identify and exclude outlier cases with significant artifacts, resulting in a refined, artifact-free dataset. In the second stage, the model was retrained on this purified dataset using an L2 loss function, which was selected for its superior ability to model fine-grained intensity relationships in the absence of noisy samples. To optimize training hyperparameters, a grid-search approach was employed to systematically explore combinations of learning rates (ranging from 1 × 10^−5^ to 1 × 10^−3^), batch sizes (2, 4, and 8), and optimizer settings (using Adam with momentum values of 0.9 and 0.99). The optimal configuration was determined based on validation performance, with a learning rate of 1 × 10^−4^, batch size of 4, and Adam optimizer (momentum = 0.9) yielding the best balance of convergence speed and model accuracy. Training was performed on a system equipped with an Intel^®^ Core™ i7-6500U CPU @ 2.5 GHz (Intel, Santa Clara, CA, USA) and an NVIDIA GeForce 940M GPU (NVIDIA, Santa Clara, CA, USA), with each epoch requiring approximately 10 h to complete.

The performance of the AC model was evaluated using the following voxel-wise metrics:

(1) $$ME=\frac{1}{vxl}\sum_{v=1}^{vxl}\left({PET}_{predicted}\left(v\right)-{PET}_{ref}\left(v\right)\right)$$

(2) $$MAE=\frac{1}{vxl}\sum_{v=1}^{vxl}\left|{PET}_{predicted}\left(v\right)-{PET}_{ref}\left(v\right)\right|$$

(3) $$RE\left(\%\right)=\frac{1}{vxl}\sum_{v=1}^{vxl}\frac{{PET}_{predicted}\left(v\right)-{PET}_{ref}\left(v\right)}{{PET}_{ref}\left(v\right)}\times 100\%$$

(4) $$ARE\left(\%\right)=\frac{1}{vxl}\sum_{v=1}^{vxl}\left|\frac{{PET}_{predicted}\left(v\right)-{PET}_{ref}\left(v\right)}{{PET}_{ref}\left(v\right)}\right|\times 100\%$$

(5) $$RE\left(\%\right)=\frac{{PET}_{predicted}\left(VOl\right)-{PET}_{ref}\left(VOl\right)}{{PET}_{ref}\left(VOl\right)}\times 100$$

(6) $$RMSE=\sqrt{\frac{1}{vxl}\sum_{v=1}^{vxl}{\left({PET}_{predicted}\left(v\right)-{PET}_{ref}\left(v\right)\right)}^{2}}$$

Additional assessment criteria included the structural similarity index (SSIM) and peak signal-to-noise ratio (PSNR), which are commonly used to evaluate perceptual image quality.

## 3. Results

The methodology employed in this study offers a robust and transparent framework to evaluate the performance of the proposed deep learning-based attenuation correction (AC) model, specifically for artifact-free ^68^Ga-PSMA PET imaging.

**Figure 2:**
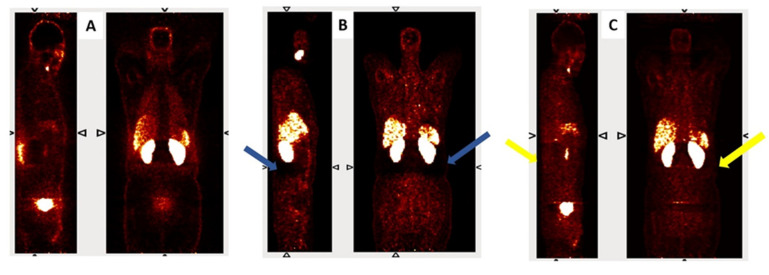
Representative coronal and sagittal views of (**A**) PET non-AC, (**B**) PET CT-AC, and (**C**) PET DL-AC-L1. Blue arrows indicate halo artifacts in the CT-AC image, while yellow arrows show partial correction in the DL-AC-L1 image.

Figure 2 presents coronal and sagittal views from a representative subject reconstructed using three different methods: non-attenuation-corrected PET (PET-non-AC), CT-based attenuation-corrected PET (PET CT-AC), and deep learning-based attenuation correction using L1 loss (PET DL-AC-L1). In the PET CT-AC image, blue arrows indicate prominent halo artifacts, especially surrounding the kidneys and bladder due to high tracer accumulation. These artifacts appear as unnatural signal voids (due to inaccurate scatter correction) and can obscure nearby lesions. In contrast, the DL-AC-L1 reconstruction (yellow arrows) demonstrates partial suppression of the halo artifact while maintaining anatomical fidelity and signal continuity. This visual comparison highlights the model’s ability to enhance image interpretability in clinically challenging regions.

**Figure 3:**
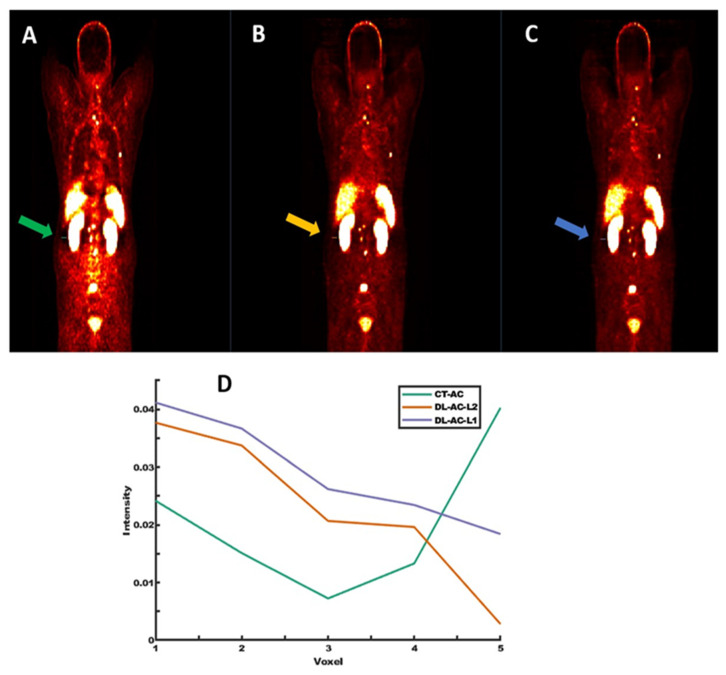
Representative images from (**A**) PET CT-AC with halo artifact, (**B**) PET DL-AC (L2 loss), and (**C**) PET DL-AC (L1 loss). (**D**) shows horizontal signal intensity profiles across the halo region, demonstrating better artifact suppression with DL-based methods (Green, orange, and purple lines correspond to the line profiles drawn on PET-CT, PET-DL-L2 loss, and PET-DL-L1 loss, respectively).

Further analysis is presented in Figure 3, where horizontal intensity profiles are drawn across the region affected by a halo artifact. These profiles compare PET CT-AC (with artifact), DL-AC predicted with L2 loss, and DL-AC predicted with L1 loss. The DL-AC-L1 model demonstrates reduced sensitivity to the artifact while preserving intensity continuity and signal uniformity. This underscores the efficacy of the model in addressing common image artifacts such as halo and motion-induced distortions.

**Figure 4:**
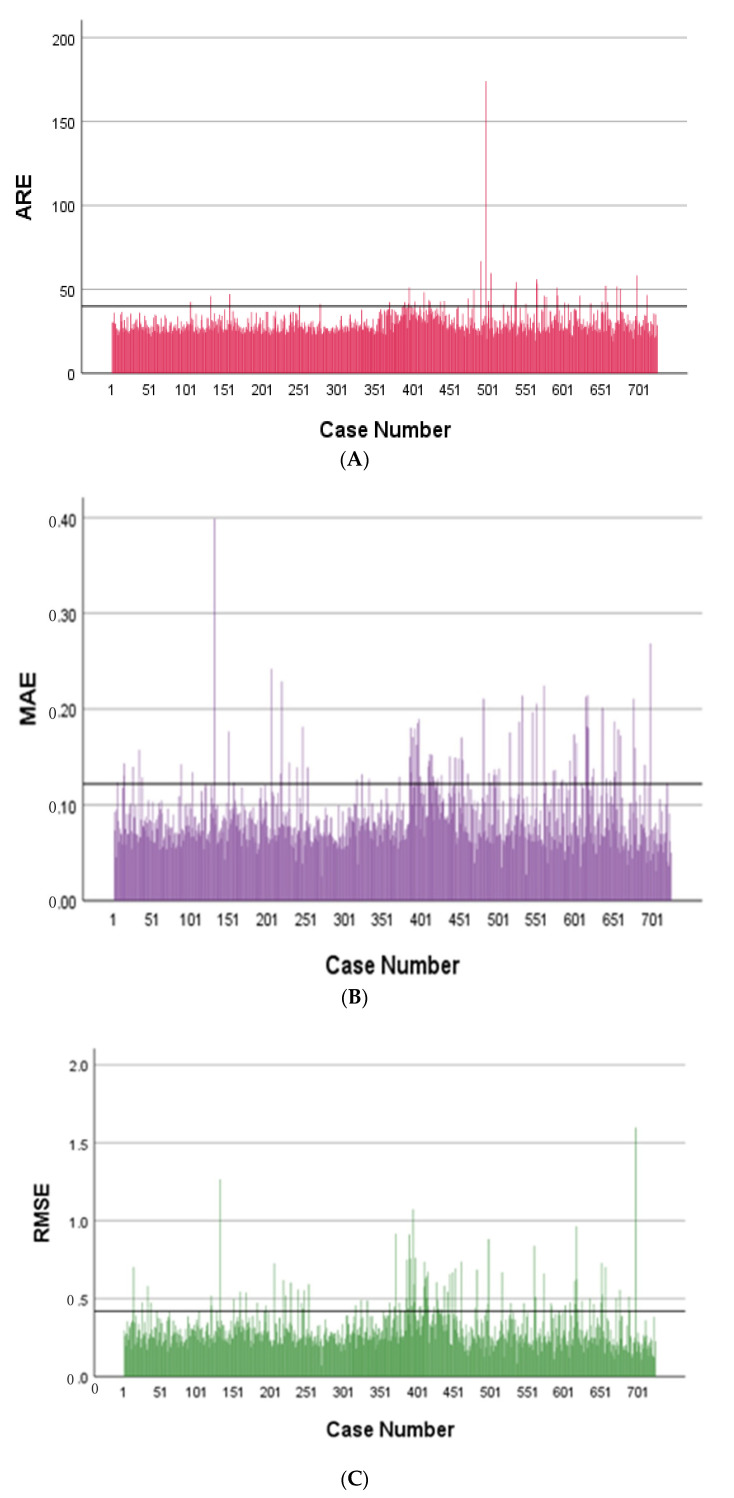
Bar plots showing: (**A**) Absolute Relative Error (ARE), (**B**) Mean Absolute Error (MAE), (**C**) Root Mean Square Error (RMSE).

A broader evaluation of the model’s performance is shown in Figure 4, which provides a quantitative comparison between CT-based attenuation correction (CT-AC) and the deep learning-based method (DL-AC), evaluated over 784 training subjects. Panels A–C display bar plots of three voxel-wise error metrics: Absolute Relative Error (ARE), Mean Absolute Error (MAE), and Root Mean Square Error (RMSE), respectively. In all cases, the DL-AC model exhibited clinically tolerable quantitative errors, demonstrating reduced quantification bias and improved consistency. It should be noted that Figure 4 shows relatively high errors in some cases, which can be attributed to artifacts in the PET CT-AC images, such as respiratory motion, halo effects, and body truncation. In contrast, PET DL-AC images are unaffected by artifacts caused by imperfect attenuation maps or scatter correction, and thus do not exhibit these issues. However, because PET CT-AC images, despite containing these artifacts, serve as the reference standard, the quantitative metrics for PET DL-AC images may indicate high errors. This discrepancy does not necessarily reflect suboptimal performance of the DL-AC approach, but rather the presence of artifacts in the reference images, which are corrected in the PET DL-AC images.

Quantitative metrics are summarized in Table 1, which compares the performance of models trained on artifact-containing data and on the purified, artifact-free dataset. The artifact-free model achieved markedly lower voxel-wise ME (−0.009 ± 0.43 counts) and MAE (0.09 ± 0.41 counts), along with a slightly reduced RMSE and competitive SSIM values. These results highlight the importance of data purification in improving generalization, reducing quantification bias, and enhancing structural fidelity.

To statistically validate this improvement, an independent samples *t*-test on MAE values resulted in a *p*-value of 0.022, demonstrating a statistically significant difference between the two groups with and without artifacts.

## 4. Discussion

A persistent challenge in ^68^Ga-PSMA PET imaging is the presence of image artifacts, most notably the halo artifact, which impairs both qualitative and quantitative interpretation. In ^68^Ga-PSMA scans, the radiotracer exhibits minimal uptake in abdominal fat and soft tissue, while it concentrates heavily within the urinary tract. This results in a stark contrast between high-uptake organs (e.g., kidneys and bladder) and surrounding tissues, creating an environment highly susceptible to photopenic halo artifacts in both PET/CT and PET/MRI modalities [6,20].

**Figure 5:**
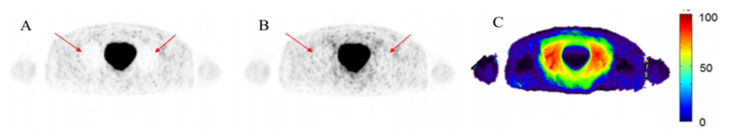
Example of the halo artifact in ^68^Ga-PSMA PET imaging. (**A**) Image with halo artifact reconstructed using standard clinical protocol (halo artifact is indicated by the red arrows). (**B**) The same scan reconstructed using modified attenuation and scatter correction, showing reduced artifact (indicated by the red arrows). (**C**) Relative voxel-wise difference image between (**A**,**B**), illustrating the artifact-induced quantification bias.

Figure 5 highlights the clinical impact of halo artifacts in ^68^Ga-PSMA PET imaging. Panel A shows a PET image reconstructed using standard clinical methods (CT-AC), where a large halo artifact appears as a signal void encircling the bladder (which is due to the inaccurate scatter correction estimated from the CT-AC map and the primary reconstruction of the PET data). Such artifacts can mask pathological uptake, reducing diagnostic confidence. Panel B displays the same scan after applying improved attenuation and scatter correction using the DL-AC method, where the halo artifact is significantly mitigated and the signal is restored in affected regions. Panel C provides a voxel-wise difference map between the two reconstructions, emphasizing the extent of quantification bias introduced by the artifact. This figure clearly demonstrates the clinical necessity for accurate artifact correction methods, particularly in the pelvic area where lesion detection is critical.

These void signals distort standardized uptake values (SUVs), leading to the misinterpretation of tumor localization and activity—particularly in proximity to the bladder and kidneys. As supported by prior findings [21], the halo artifact is primarily the result of inaccurate scatter correction during PET image reconstruction. Traditional scatter correction methods, such as single scatter simulation followed by tail fitting, are vulnerable to failure under high-contrast conditions [22].

Alternative strategies, such as absolute scaling algorithms, have been introduced to address these limitations in ^68^Ga-PSMA imaging [23]. Moreover, deep learning (DL) has emerged as a promising solution, enabling the reconstruction of artifact-corrected images through data-driven learning from unpaired or corrupted data. For instance, Whiteley et al. [24] demonstrated that DL-based PET reconstruction models can generate images comparable to full-count datasets and superior to conventional reconstructions. Similarly, Gjesteby et al. [25] showed that DL could mitigate metallic artifacts in CT images.

One of the well-known artifacts in PET/CT imaging is the mismatch between PET and CT images due to the patient’s breathing, also known as the motion artifact. Typically, CT acquisition occurs within a few seconds, while PET acquisition takes several minutes (2–3 min per bed position) [26], during which the patient breathes normally. This time discrepancy often leads to misalignment artifacts, especially at the thoracoabdominal interface, where lesions from the upper liver may appear in the lower right lung and vice versa, causing diagnostic inaccuracies.

The halo artifact is also characterized by a strong, uninterpretable signal void surrounding regions with high tracer uptake [21,27]. This artifact commonly appears around the bladder and kidneys, where ^68^Ga-PSMA tends to accumulate intensely. If scatter correction fails during PET image reconstruction, it results in photopenic areas around these high-uptake regions, degrading image quality and affecting quantitative accuracy [21].

Another common issue is the truncation artifact, which occurs when the patient’s body diameter exceeds 50 cm—the maximum field-of-view (FOV) of standard CT scanners [28]. As a result, part of the anatomical data needed for attenuation correction is missing. Metallic artifacts, arising from prosthetic implants such as hip replacements, pose further challenges. Metallic materials absorb a significant portion of X-ray photons during CT, causing hyperdense streaks that propagate into the attenuation map and introduce inaccuracies in nearby PET signal quantification [28].

These artifacts highlight inherent limitations in CT-based attenuation and scatter correction. To overcome them, AI-assisted image reconstruction techniques have been introduced [3]. McMillan and Bradshaw [29] observed that the ability of AI-based approaches to obviate the need for an additional CT or transmission image greatly improves the capability of existing equipment. Similarly, Arabi et al. and Hashimoto et al. [30,31] emphasized the robust performance of Deep Learning-based Attenuation and Scatter Correction (DL-AC) across different radiotracers.

The Deep-JASC framework developed by Shiri et al. [32] demonstrated that deep learning can jointly correct attenuation and scatter without requiring CT or MRI inputs, making it well-suited for standalone PET scanners and PET/MRI systems. In one of their studies, they reported ME = 0.02 ± 0.05, MAE = 0.20 ± 0.07, RE = −1.32 ± 2.5%, ARE = 10.0 ± 4.5%, and SSIM = 0.98 ± 0.01 in the hold-out test set, affirming high fidelity in artifact correction.

In our work, we identified various artifacts in CT-ASC images and implemented a novel artifact disentanglement framework using deep learning. The proposed algorithm demonstrated the ability to effectively correct halo, truncation, motion, mismatch, and metallic artifacts in both PET/CT and PET/MRI modalities. Moreover, it can serve as an efficient and fast quality assurance tool for routine clinical deployment.

The halo artifact was operationally defined as a volume of consecutive zero-valued voxels located around the kidneys or bladder. These halos were semi-automatically segmented for each PET image using the MITK image-processing platform [33], providing consistency in the evaluation process.

Moreover, Li et al. [34] applied a deep convolutional encoder–decoder (CED) network based on a U-Net architecture for whole-body PET attenuation correction and reported an ARE of approximately 30% for 10 subjects. Yang et al. [35] introduced 3D generative adversarial networks (3D-GANs) for attenuation and scatter correction in whole-body PET and reported average ME and MAE values of 2.49% ± 7.98% and 16.55% ± 4.43%, respectively, across 30 patients.

More recently, Dong et al. [36] proposed a structure-independent deep learning approach for attenuation correction. Their method achieved ME values of 13.84% ± 10.11%, 13.42% ± 10.13%, and −17.02% ± 11.98% in the lungs for U-Net, GAN, and cycle-GAN, respectively. In whole-body imaging, the corresponding ME values were 2.05% ± 2.21%, 2.25% ± 1.93%, and 0.62% ± 1.26%, demonstrating that high performance is achievable even without structural input, provided the training dataset is sufficiently large and clean.

The results of our study highlight the significant improvements achieved through our proposed deep learning-based attenuation correction (AC) framework in ^68^Ga-PSMA PET imaging. Visual evaluation of coronal and sagittal slices comparing PET CT-AC with PET DL-AC (trained using the L1 loss) revealed a marked reduction in halo artifacts. Additionally, horizontal intensity profiles drawn across halo-affected regions showed that the DL-AC model had a higher capacity to suppress photopenic zones.

Despite the L1 loss function’s robustness to artifacts, the final performance evaluation was conducted using models trained with the L2 loss function, which demonstrated better convergence for cleaned datasets. The final reported results—ME = −0.009 ± 0.43 SUV, MAE = 0.09 ± 0.41 SUV—clearly outperformed the model trained on the original unfiltered dataset.

Moreover, external validation reinforced these findings, with consistently improved MAE, RE, RMSE, and SSIM across all test subsets, further affirming the generalizability and robustness of the proposed purification-based training strategy.

This advancement in attenuation correction performance holds promise not only for ^68^Ga-PSMA PET but also for other radiotracers and clinical applications where artifact-induced errors limit diagnostic confidence. Ultimately, our approach supports the broader integration of deep learning models into clinical nuclear medicine, contributing to more accurate, reliable, and artifact-resilient quantitative PET imaging.

The integration of white-box uncertainty metrics, such as voxel-wise variance maps, into our DL-AC framework for ^68^Ga-PSMA PET imaging could significantly enhance its clinical utility and reliability. By providing a spatially resolved measure of the model’s confidence in its predictions, these uncertainty maps would enable clinicians to assess the trustworthiness of the generated PET DL-AC images, particularly in regions prone to artifacts like halo effects, respiratory motion, or truncation errors, which are prevalent in ^68^Ga-PSMA PET due to high-contrast tracer uptake in the kidneys and bladder. For instance, high uncertainty in areas affected by halo artifacts could alert clinicians to potential misinterpretations, such as obscured lesions near the bladder or kidneys, prompting the use of alternative imaging or correction strategies to ensure accurate diagnosis and staging of prostate cancer. Furthermore, these maps could serve as a quality assurance tool, facilitating the identification of images with significant artifacts during routine clinical workflows, thereby reducing the risk of diagnostic errors and improving quantitative accuracy of SUVs. While our current deterministic ResNet model effectively mitigates artifacts through data purification, incorporating uncertainty quantification represents a promising future direction to further enhance the robustness and interpretability of AI-driven PET imaging, aligning with the broader goal of improving diagnostic confidence in nuclear medicine.

This study has several limitations. First, clinical studies lack a ground truth, so the evaluation of artifact reduction in PET DL-AC images relied solely on visual assessment. To address this limitation, dedicated phantom studies with known ground truth could be conducted to objectively evaluate the performance of the DL-AC method. Additionally, comprehensive qualitative analysis by nuclear medicine specialists, comparing the quality of PET CT-AC and DL-AC images, could provide insights into the clinical value of the proposed DL-AC method. Second, this study utilized data from a single center and PET/CT scanner. Conducting a similar study with a multi-center dataset would enhance the generalizability of the findings. Third, the dataset in this study was imbalanced, with varying numbers of PET images containing no artifacts versus those with specific artifacts, such as halo effects or respiratory motion, which were more prevalent. This imbalance may skew results. Evaluating the method on a balanced dataset could improve the model’s performance for less common artifacts. Finally, the absence of an ablation study focusing on specific artifacts, such as halo effects, limits the ability to assess the DL-AC method’s performance for individual artifact types. Conducting such a study could provide deeper insights into the model’s effectiveness and robustness across different artifact scenarios.

## 5. Conclusions

This study presents an effective deep learning-based framework for image-domain attenuation correction in ^68^Ga-PSMA PET imaging, emphasizing the importance of artifact-free training data. By purifying the dataset through self-inference and empirical error analysis, we significantly enhanced the accuracy and robustness of the AC model. Quantitative evaluation demonstrated marked improvements in key performance metrics across internal and external test sets. Beyond attenuation correction, the methodology offers a scalable solution for artifact reduction in whole-body PET imaging and underscores the value of data quality in training high-performing AI models for clinical nuclear medicine.

## Figures and Tables

**Table 1:** Statistical analysis of image quality metrics. Comparison of models trained on datasets with and without artifacts. Evaluation includes ME, MAE, RE%, RMSE, and SSIM, with PET CT-AC used as the reference.

Methods	ME (Counts)	MAE (Counts)	RE (%)	RMSE (SUV)	SSIM
DL-AC-L2 (With artifact)	−0.11 ± 0.42	0.91 ± 0.29	−2.47 ± 10.10	0.30 ± 0.12	0.97 ± 0.03
DL-AC-L2 (Artifact-free)	−0.009 ± 0.43	0.09 ± 0.41	1.55 ± 16.78	0.26 ± 0.16	0.96 ± 0.03

## Data Availability

The datasets generated and analyzed during the current study are available from the corresponding author upon reasonable request, subject to institutional approvals.
